# Relationships among total mixed ration nutritional components and reproductive performance in high-producing dairy herds

**DOI:** 10.3168/jdsc.2022-0265

**Published:** 2023-01-12

**Authors:** Carlos E.C. Consentini, Alexandre H. Souza, Roberto Sartori, Paulo D. Carvalho, Randy Shaver, Milo C. Wiltbank

**Affiliations:** 1 Department of Animal Science, ESALQ, University of São Paulo, Piracicaba, SP, Brazil, 13418-900; 2 Department of Animal and Dairy Sciences, University of Wisconsin-Madison, Madison 53706; 3 Cargill Animal Nutrition and Health, Campinas, SP, Brazil, 13091-611

## Abstract

The main objective of the present study was to determine whether composition of total mixed ration influences reproductive performance in high-producing commercial dairy farms. Dairy producers and nutritional consultants from 48 dairy farms located in Wisconsin agreed to provide reproductive data and dietary information on high milk production pens during the main breeding period for the previous 12 mo. Dietary components (percentage of dry matter) were crude protein (CP), rumen degradable (RDP) and undegradable (RUP) protein, neutral detergent fiber (NDF), nonfiber carbohydrates (NFC), starch, and fat. Reproductive data were service rate (SR), overall pregnancy per artificial insemination (P/AI) and P/AI at the first service, 21-d pregnancy rate (PR), days open, and percentage of cows pregnant by 150 d in milk (PREG150). Participating herds had lactating Holstein cows (range = 143 to 2,717) housed in freestall facilities. Statistical analyses were performed with CORR and GLIMMIX of SAS (SAS Institute Inc.). Daily average milk production of herds was 38.9 ± 0.60 kg/d (30.0 to 50.4 kg/d). Overall SR was 58.5% (39–73) and P/AI was 36.1% (22–49). Overall 21-d PR was 20.3% (10–42%). Correlation between SR and PR was 0.59, whereas correlation of overall P/AI and P/AI at first service with PR were both 0.72. Similarly, for PREG150, correlation with overall P/AI (0.63) and P/AI at first service (0.66) were greater than with SR (0.48). There was large variation in diet composition, with CP varying from 16.0 to 18.7%, NDF from 24.9 to 35.1%, NFC from 31.7 to 46.6%, starch from 20.1 to 30.8%, and fat from 3.1 to 6.7%. Overall, there were no detectable associations of CP, RDP, and RUP with reproductive measures. The strongest relationship was a decrease in reproductive performance with increasing dietary NFC including overall P/AI (−0.48), P/AI at first service (−0.51), and PREG150 (−0.33). Starch also had a negative relationship with P/AI at first service (−0.35). Conversely, greater NDF was positively associated with P/AI at first service (0.34). Fat content was also positively associated with P/AI at first service (0.34). When NFC was divided in tertiles (<40, 40 to 42.2, and >42.2% NFC), the highest tertile had lower overall P/AI (39 vs. 36 vs. 31%), P/AI at first service (43 vs. 40 vs. 33%), and PREG150 (54 vs. 53 vs. 47%). In conclusion, farms with greater dietary NFC may have compromised reproductive performance. Correspondingly, herds with greater NDF content may achieve high milk production with potentially positive associations with reproduction. Other relationships of dietary components on reproduction were not as obvious in this herd-level analysis.

## Highlights

• High NDF content in TMR diets increases fertility at first postpartum service.
• High NFC content in TMR diets decreases fertility and reproductive performance.
• Fertility (overall and at first AI) has a greater impact on reproduction than service rate.

## Graphical Abstract

**Figure:**
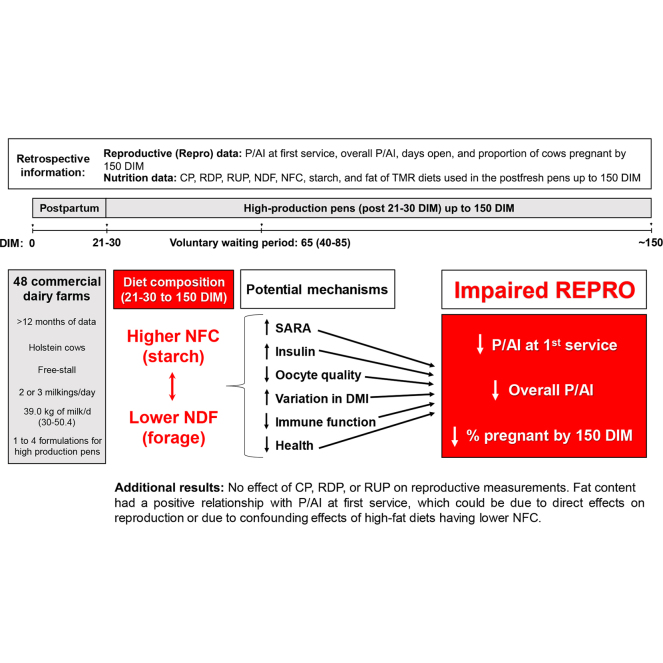
**Summary:** The study evaluated the effect of total mixed ration (TMR) composition on reproductive performance of highproducing commercial dairy herds. Crude protein (CP) and rumen degradable (RDP) and undegradable (RUP) protein had no effect on reproduction. Fat content was positively associated with pregnancy per AI (P/AI) at first service. The key results were related to the effects of nonfiber carbohydrates (NFC) and neutral detergent fiber (NDF), in which higher NFC was associated with lower overall fertility and P/AI at the first service, in addition to fewer cows pregnant by 150 days in milk. The results of this study suggest that farms with higher dietary NFC, particularly during early lactation, may have compromised reproductive performance. On the other hand, herds with greater NDF content potentially have positive effects on reproduction. Other effects of dietary components on reproduction were not as obvious in this herd-level analysis.

Reproductive performance is an important determinant of dairy herd efficiency with an optimized calving interval increasing milk production, subsequent reproductive performance, and farm profitability (Middleton et al., 2019). Efficiency of reproduction in high-producing dairy cows is affected by numerous factors including heat stress (Baruselli et al., 2020), BCS and BCS changes (Carvalho et al., 2014), health problems (Carvalho et al., 2019), timed artificial insemination (**TAI**) programs (Consentini et al., 2021), and nutrition (Rodney et al., 2018). This study focused on the impact of specific nutritional components in the TMR on various measures of reproductive performance in well-managed Midwestern dairy farms.

Previous studies have focused on the impact on reproduction of specific nutritional manipulations such as acidogenic diets (Santos et al., 2019), supplementation of specific fatty acids (Rodney et al., 2015), AA such as methionine and methyl-group donors like choline (Zhou et al., 2016), and manipulation of dietary energy and starch sources (Albornoz and Allen, 2018; Cardoso et al., 2020). Despite the potential impact of nutrition on dairy cow reproduction, it is challenging to perform valid nutrition-reproduction experiments due to the necessity for continuous manipulation of the diet in a large number of animals to validly quantify changes in binomial fertility values such as pregnancy per AI (**P/AI**). Thus, the relationships among key components of the TMR, which have known effects on milk production, have not been systematically connected to reproductive performance. The main objective of the present study was to determine whether composition of TMR influences reproductive measures on high-producing commercial dairy farms. The approach was to use dietary data from nutrition consultants, such as concentrations of protein, fiber, carbohydrate, and fat in the TMR, and to correlate this information with reproductive data collected during the same time period. This experimental approach did not allow testing of specific dietary components but was designed to identify key components of the TMR that may affect reproduction to help direct future manipulative studies on nutrition-reproduction interactions in high-producing dairy cows.

Data from 48 commercial dairy farms located in Wisconsin were retrieved directly from nutrition consultants on each dairy to create the dietary component database. All participating herds had more than 100 (range = 143 to 2,717) lactating Holstein cows housed in freestall facilities. The farmers consented to provide their complete diets and accurate production and reproductive records with archive files for the previous 12 mo that matched the period of the TMR. Nutritional information included all dietary ingredients and nutrient compositions of the diets for the high-production cow pens post 21 to 30 DIM. Thus, the diet information retrieved from all herds coincided with the main breeding period after calving, which started after the end of the voluntary waiting period and up to ~150 DIM. This research used only nutritional and reproductive records from dairy farms and does not contain any studies with human or animal subjects, so it did not require Institutional Animal Care and Use Committee or Institutional Review Board approval.

A total of 64 diet ingredients were identified including forage and concentrate sources, fat and AA supplements, byproduct feeds, minerals, and vitamins. Complete dietary composition was analyzed by each nutrional consultant at multiple times during the experimental period with mean values obtained for each farm on the content (percentage of DM) of CP, RDP, RUP, NDF, NFC, starch, and fat.

The reproductive data were retrieved by the same technician from the Dairy Comp 305 (Valley Ag Software) and PCDART herd management software (Dairy Records Management Systems), and excluded “do not breed” cows. The main data retrieved were the percentage of TAI used for first service and for all AI, service rate (**SR**), overall P/AI and P/AI at the first service, 21-d pregnancy rate (**PR**), days open (**DOPN**), and percentage of cows pregnant by 150 DIM (**PREG150**).

Statistical analyses were performed using the Statistical Analysis System (SAS, version 9.4 for Windows, SAS Institute Inc.). Data were tested for normality of residuals with the Shapiro-Wilk test, using the UNIVARIATE procedure of SAS. Correlation tests between dietary components and reproductive measures were performed with the CORR procedure, and logistic regressions were performed using the GLIMMIX procedure fitting a Gaussian distribution. For some variables with significant correlations, the intercept and slope of equations were obtained using the option solution in the GLIMMIX procedure. Additionally, the option ddfm = kenwardroger was included in the model statement to adjust the degrees of freedom for variances. In addition to the logistic regressions performed considering the diet components as continuous variables, tertiles were created according to the level of the component, for example NDF and NFC, to study the effect of those components as class independent variables.

Tukey honest significant difference post hoc test was performed for mean separation. Values are presented as mean ± standard error of the mean. Significant differences were declared when *P* ≤ 0.05, whereas tendencies were considered when 0.05 < *P* ≤ 0.10.

Daily average milk production of the herds was 38.9 ± 0.60 kg/d, varying from 30.0 to 50.4 kg/d. The average milk fat and protein percentage and SCC were 3.67 ± 0.03, 3.05 ± 0.01, and 246,500 ± 13,999, respectively, and there was no effect (*P* > 0.10) of herd size on any of these milk parameters.

The voluntary waiting period was 65 DIM, on average, ranging from 40 to 85 DIM. For reproductive management, most of the herds used exclusively TAI for first service, with an average across herds of 80% (25–100) for the first service, and the average for all inseminations of 65% (15–99). As expected, the SR (58.5% overall, ranging from 39 to 73) increased as the percentage of TAI use increased. However, interestingly, the percentage of TAI use for first service had a stronger relationship (r = 0.53; *P* = 0.0003) with SR than overall TAI use (r = 0.33; *P* = 0.03).

The overall P/AI was 36.1% (22–49), with primiparous cows having 19% greater fertility than multiparous cows (40.4 vs. 34.0%). The overall fertility at first service was 39.7% (20–51), with a P/AI of 45.9% in primiparous and 36.2% in multiparous cows. Overall 21-d PR from all farms was 20.3%, ranging from 10 to 42%. Percentage of cows pregnant by 150 DIM and overall DOPN was 52% (30–75) and 129 d (96–189), respectively. The 21-d PR and PREG150 are important measures of reproductive efficiency, and both are influenced by SR and P/AI. Interestingly, in our database, overall P/AI and P/AI at first service had greater relationships with 21-d PR and PREG150 compared with SR. The correlation coefficient between SR and PR was 0.59 (*P* < 0.0001), whereas the correlations between overall P/AI and P/AI at first service with PR were both 0.72 (*P* < 0.0001). Similarly, for PREG150, the correlations with overall P/AI (0.63; *P* < 0.0001) and P/AI at first service (0.66; *P* < 0.0001) were greater than with SR (0.48; *P* = 0.001). As discussed previously, reproductive efficiency is associated with the efficiency, timing, and fertility to the first and later AI programs (Giordano et al., 2012). The stronger association of P/AI with reproductive performance compared with SR highlights the importance of using programs to increase SR (such as use of TAI), but also using programs and management to maximize fertility (Consentini et al., 2021), for example, implementing fertility programs at first service (Fricke and Wiltbank, 2022) since fertility at first AI is a major driver of reproductive performance.

Regarding general nutritional information, the percentage of forage in the diets varied from 48 to 60% (average = 56.1%), and the variation in the main components of the diet is depicted in Figure 1. As shown, there is considerable variation in TMR among herds, particularly in forage, starch, NDF, and NFC content of the diets. The variation in vitamin content in the TMR diets was surprisingly large, with vitamin A ranging from 93,000 to 401,000 IU, vitamin D from 28,700 to 72,800 IU, and vitamin E ranging from 460 to 2,868 IU per cow per day. Several factors could influence ingredients used within a farm and, thus, TMR composition, such as quality and type of forage, price and availability of ingredients, and the necessity or desire to include a particular ingredient by a nutritionist or dairy producer.

**Figure 1:**
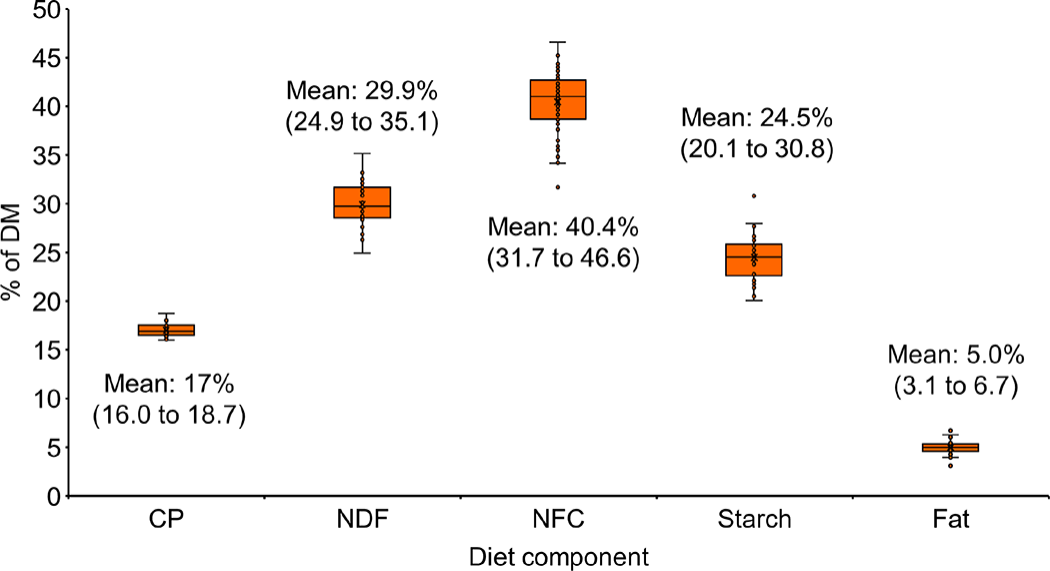
Variation in dietary components (% of DM) among high-producing dairy herds. The dots represent the real values for the dietary component on each individual farm with the error lines showing the range without the extreme values. The box shows the median (middle line) and 2 quartiles.

There was no correlation between NFC (r = −0.14; *P* = 0.32), NDF (r = 0.08; *P* = 0.57), CP (r = 0.06; *P* = 0.70), RDP (r = 0.04; *P* = 0.79), RUP (r = 0.02; *P* = 0.98), starch (r = −0.22; *P* = 0.22), or fat (r = 0.23; *P* = 0.12) content of the diets in high-production pens with herd average milk production. Moreover, the variation in NFC (32 to 47) and starch (20 to 31) among the farms in this study is within the range of NFC and starch values reported for high-producing cows (NRC, 2001; National Academies of Sciences and Medicine, 2021). Thus, it may be possible for dairy herds to feed well-formulated diets with controlled starch and NFC levels, with adequate forage and nonforage ingredients, and still achieve high milk production. Various factors influence milk production, many of which were not controlled or evaluated in the present study. However, these results are encouraging in terms of attempting to better understand variation in the main components of the diets among farms and their influence on milk production. For instance, it would be interesting to experimentally evaluate if controlled levels of NFC and higher forage NDF would allow high milk production while improving reproduction.

The relationships between various aspects of the TMR and 3 measures of reproductive efficiency are in Table 1. The 3 measures of reproductive performance were chosen because there was no correlation between any of the dietary components and SR and correlations with PREG150 were very similar to correlations with PR. Overall, there were no detectable associations of dietary protein, expressed as CP, RDP, or RUP, on these reproductive measures across the dairy herds. The strongest relationship was found for NFC with decreasing reproductive performance with increasing NFC. This negative relationship of NFC was significant for P/AI at first service, overall P/AI, or percentage pregnant at 150 DIM. Conversely, greater NDF was associated with greater P/AI at first service (Table 1).

**Table 1:** Correlation between dietary components and reproductive measures in high-producing commercial dairy herds^1^

Item (% of DM)	Reproductive measurement		
	P/AI at first service	Overall P/AI	Pregnant by 150 DIM
CP	0.05 (0.73)	0.16 (0.31)	−0.12 (0.45)
RDP	−0.11 (0.48)	−0.03 (0.85)	−0.16 (0.32)
RUP	0.23 (0.14)	0.26 (0.10)	0.06 (0.70)
NDF	0.34 (0.03)	0.25 (0.11)	0.11 (0.48)
NFC	−0.51 (0.0005)	−0.48 (0.001)	−0.33 (0.03)
Starch	−0.35 (0.05)	−0.20 (0.28)	−0.16 (0.38)
Fat	0.34 (0.02)	0.24 (0.12)	0.24 (0.17)

1 The table shows correlation coefficient (r) and *P*-value (in parentheses). P/AI = pregnancy per artificial insemination.

Figure 2 illustrates the relationships between NDF and NFC with reproductive measures. As shown, NDF is positively associated with P/AI at first service either when comparisons were made with all individual herd data or if herds were divided by tertiles for NDF and compared with reproductive measures. Conversely, there was a strong negative association between all 3 measures of reproductive performance with NFC, either on an individual herd basis or when herds were divided by tertiles (Figure 2).

**Figure 2:**
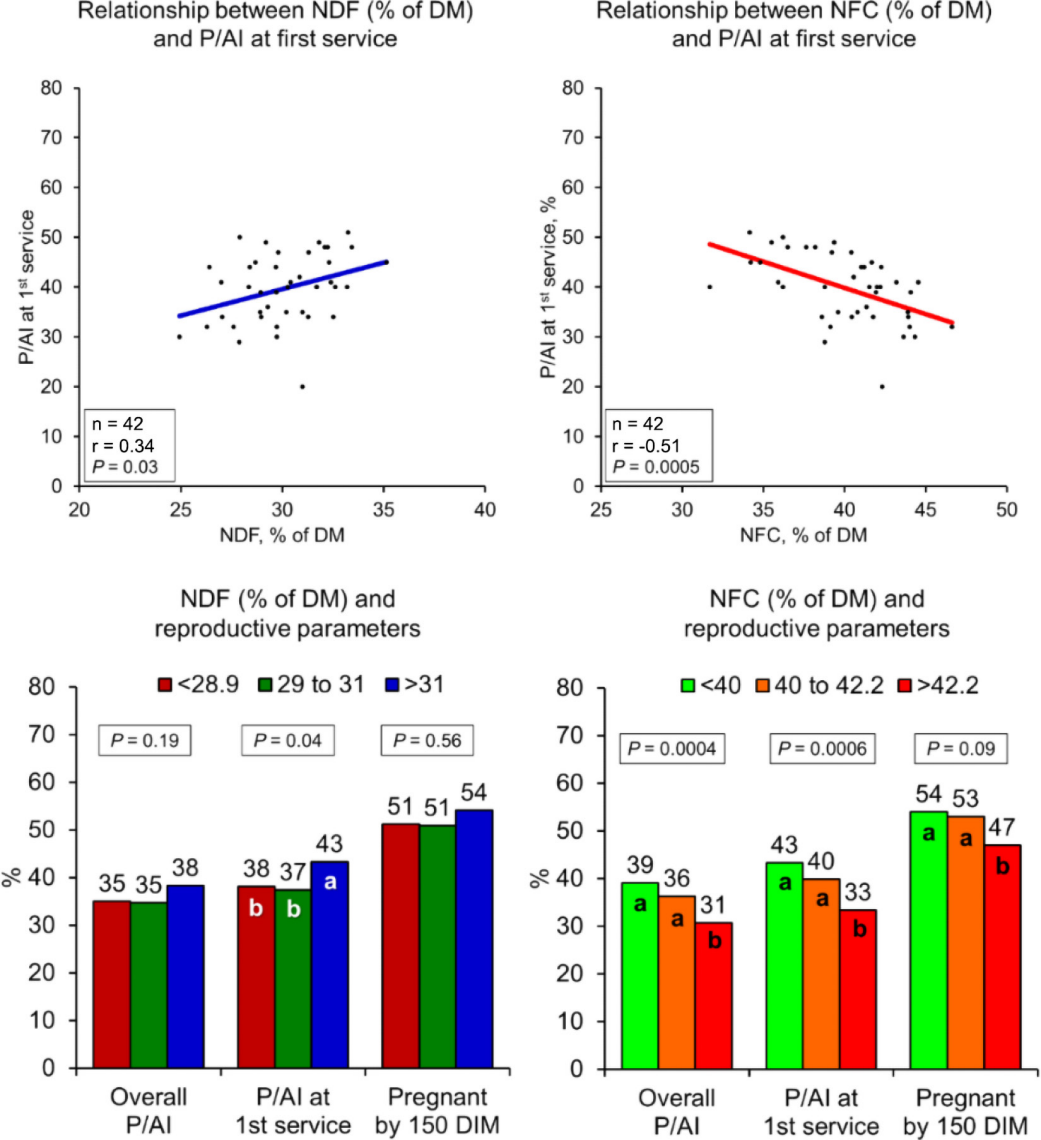
Relationship between dietary levels of NDF and NFC and reproductive outcomes in high-producing commercial dairy herds. P/AI = pregnancy per artificial insemination. Different letters (a, b) indicate differences (*P* < 0.05) within a reproductive outcome.

The methodology used in the present study does not allow us to determine the reasons that specific dairy herds had greater or less NFC in their diets or the mechanisms that produced the negative correlations with overall P/AI (−0.48), P/AI at first service (−0.51), and PREG150 (−0.33). When the effect of starch level of diets was evaluated, similar to NFC, starch had a negative relationship with P/AI at first service (−0.35; *P* = 0.05), but had no detectable associations with overall P/AI or PREG150 (Table 1). Previous research has shown that high starch diets lead to increased insulin and this are associated with reduced fertilization of oocytes, increased degeneration of embryos, and speculatively, these effects may underlie the observed reduction in herd fertility observed in this study (Bender et al., 2014; Wiltbank et al., 2014). Alternative explanations could be the decrease in DMI associated with high starch diets (Albornoz and Allen 2018), upregulation of genes associated with inflammation (Khafipour et al., 2009; Albornoz et al., 2020), or occurrence of SARA due to high starch diets (Khorrami et al., 2021). Thus, these negative aspects of high NFC diets could impair reproductive performance, in spite of potential benefits of increased dietary energy coming from NFC. Figure 2 shows the negative associations of NFC and reproductive performance, particularly in herds with higher NFC, as P/AI decreased from 43 to 33% (>20% reduction in relative P/AI) with corresponding decreases in overall P/AI and PREG150. Conversely, the positive association of NDF with better reproduction could, speculatively, be the reduction in NFC, thereby reducing some potential negative mechanisms that are discussed above.

The effect of fat on reproduction has been extensively studied in past research and our study also found a positive correlation of percentage fat in the TMR with P/AI at first service. This could be due to multiple reasons. First, when dietary starch and NFC are reduced, fat may be added to the diet to increase the energy content of the diet. As expected, level of fat had a negative correlation with NFC in our database (−0.49; *P* = 0.0004). Second, several studies have evaluated relationships of dietary fat with milk production, health, and reproduction through studies that supplemented cows during the transition period, early lactation, or both (Rodney et al., 2018). Generally, there is a positive association of fat supplementation, particularly UFA, on health, follicle and corpus luteum development, and pregnancy outcomes (Santos et al., 2008). For instance, Sinedino et al. (2017) supplemented cows after the transition period (from 27 to 147 DIM) with docosahexaenoic acid and reported better cyclicity and greater P/AI at first service, particularly in primiparous, and greater overall P/AI. In another study, cows supplemented with fish oil during the breeding period (30 to 160 DIM) had greater overall P/AI on d 60 and lower pregnancy loss (Silvestre et al., 2011). Altogether, the current findings argue for a positive association of fat supplementation with reproduction, although it is hard to determine the mechanisms that produce this association.

The lack of an association of dietary protein with any of our reproductive measures is interesting; however, it should be noted that the variation in dietary CP, RDP, and RUP levels was not as large as variation in other components of the diets. Some previous studies have noted a negative association of BUN or MUN on reproduction (Webb and de Bruyn, 2021). The observed MUN can be influenced by CP, RDP, and RUP levels, as well as the quality of protein, and energy in the diet. We expected no association of protein with reproduction, since modern well-formulated diets generally do not have issues with elevated MUN. Consistent with our results, a previous meta-analysis also reported no effect of CP, RDP, or RUP on P/AI or interval from calving to pregnancy (Rodney et al., 2018).

Finally, the limitations of this type of study need to be emphasized. There are numerous dietary and management factors that can greatly affect reproductive performance such as cow comfort, reproductive program, pen size, stocking density, and type of housing to name just a few factors that could cause variation in reproductive performance between dairies (Chebel et al., 2016; Wang et al., 2016; Jensen and Proudfoot, 2017). Some other management factors with potentially important effects on fertility such as homogeneity of TMR provided within pens or across days, consistency in feeding times, or even feeding deviations due to external factors (such as rainfall or other weather event) were not taken into account in this study. In addition, other characteristics of the ingredients and diets that were not evaluated in this study could influence DMI, energy balance, milk production, behavior, and reproduction. For example, fat supplementation in our database was not detailed in depth. It is known that fat supplementation can affect NDF digestibility, DMI, milk production, and NDF content based on type of fatty acids (saturated, n-3, n-6), level of inclusion, and period of lactation (Piantoni et al., 2015; Weld and Armentano, 2017; de Souza et al., 2019, 2021), and these factors could change reproductive performance. Similarly, the quality and physical characteristics of the forage sources could differ substantially among farms, and it is reported that type of forage, fiber content and digestibility, and particle size influence DMI, and behavior patterns such as eating, rumination, chewing, and resting (Jiang et al., 2017; Grant and Ferraretto, 2018). Finally, negative energy balance and BCS changes during the transition period and early lactation are likely to differ substantially among farms and it is well established that BCS changes during early lactation dramatically affect health, fertility at first service, and reproductive performance (Carvalho et al., 2014; Barletta et al., 2017). Thus, since our analysis was based on differences in reproductive performance between different dairy herds that were not controlled for numerous confounding factors, the results should not be used as definitive proof for any specific theory. Instead, these results can be used as the rationale for further studies on the critical topic of the effects of nutrition on reproduction in lactating dairy cows.

In conclusion, the results of this study suggest that farms with greater dietary NFC, particularly during early lactation, may have compromised reproductive performance, such as decreased P/AI at first service, lower overall fertility, and fewer cows pregnant by 150 DIM. On the other hand, in the herds used in this study, greater NDF content was positively associated with reproduction. Other associations of dietary components with reproduction were not as obvious in this herd-level analysis.
